# Unexpectedly high renal pathological scores of two female siblings with Fabry disease presenting with urinary mulberry cells without microalbuminuria

**DOI:** 10.1016/j.ymgmr.2022.100874

**Published:** 2022-04-22

**Authors:** Natsuo Yamada, Hirofumi Sakuma, Mitsuru Yanai, Ayana Suzuki, Keisuke Maruyama, Motoki Matsuki, Naoki Nakagawa

**Affiliations:** a Division of Cardiology, Nephrology, Pulmonology and Neurology, Department of Internal Medicine, Asahikawa Medical University, Asahikawa, Japan; b Department of Pathology, Sapporo Tokushukai Hospital, Sapporo, Japan

**Keywords:** Fabry disease, Urinary mulberry cells, Renal pathology

## Abstract

We describe the cases of 47- and 45-year-old sisters who were diagnosed with Fabry disease by genomic analysis. Although the only abnormal finding was the presence of mulberry cells in their urinary sediment, the renal pathological scores, which were evaluated by light and electron microscopy, were unexpectedly very high due to severe accumulation of globotriaosylceramide in the glomerular podocytes and tubular epithelial cells. Nephrologists and laboratory technicians should recognize the importance of screening for mulberry cells during urinalysis as this is a simple, inexpensive, and non-invasive method for early diagnosis, leading to early treatment of Fabry disease.

## 1 Introduction

Fabry disease is an X-linked lysosomal storage disorder caused by abnormalities in the *α-galactosidase* (*Gal*) *A* gene (*GLA*) (MIM:300644), which leads to reduced activity of the lysosomal enzyme, α-galactosidase A (α-Gal A) [1,2]. This in turn leads to classic early manifestations (acroparesthesia, clustered angiokeratoma, cornea verticillata, hypohidrosis), and multiorgan diseases affecting the heart [3,4], kidneys [5,6], and brain [7,8]. The treatment of patients with Fabry disease primarily focuses on replacing the missing or deficient enzyme (α-Gal A) with enzyme replacement therapy (ERT), as well as treating the various symptoms and disease complications [9]. Furthermore, an oral pharmacological chaperone can now be used instead of ERT as an option only in patients with amenable genetic variants (present in 35–50% of patients) that allow for a substantial increase in enzyme activity with this medication [9], although many patients with amenable mutations in the *GLA* do not have classical disease, nor serious late onset Fabry disease.

Lyso-Gb3 (globotriaosylsphingosine), the deacylated form of globotriaosylceramide (Gb3), is currently measured in plasma and urine as a biomarker of classic Fabry disease [10,11]. Recently, urinary mulberry cells have come into focus as an inexpensive, noninvasive, and useful diagnostic tool for the early diagnosis of Fabry disease [12,13]. Urinary mulberry cells are distal tubular epithelial cells and/or podocytes in which Gb3 has accumulated; they are the characteristic feature of Fabry disease. Moreover, urinary mulberry bodies are a component of mulberry cells that can be distinguished easily from fat particles by their inner lamellar appearance [14]. At present, there are few reports on the association between the presence of urinary mulberry cells and renal pathological findings based on the scoring system of the International Study Group of Fabry Nephropathy (ISGFN) [15]. Here, we describe the unexpectedly high renal pathological scores of two female siblings with undiagnosed Fabry disease that presented with urinary mulberry cells but without microalbuminuria or proteinuria.

## 2 Cases

### 2.1 Case 1: Elder sister

A 47-year-old Japanese woman had periodic crises of severe pain in the extremities (acroparesthesia) in childhood and has suffered from hypohidrosis since childhood. Her mother had cardiac failure and died at 60 years of age. After her brother was diagnosed with Fabry disease based on the detection of urinary mulberry bodies [16], she asked her attending physician to check her for Fabry disease, and the patient was referred to our hospital for assessment. The patient was diagnosed with Fabry disease based on decreased α-Gal A activity (<1.0 pmol/h/disk; cutoff value <7.0 pmol/h/disk in females) and the presence of the same mutation as her brother, c.723dupT in exon 5 of *GLA* [4]. Hypohidrosis, and cornea verticillata were present but angiokeratomas were absent at the time of diagnosis.

**Fig. 1:**
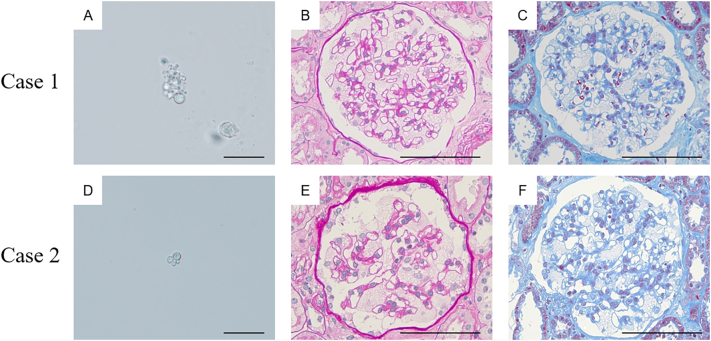
Morphology of Mulberry cells in the urine sediment of case 1 (A) and 2 (D) (bar = 20 μm). Representative light microscopy images of siblings with Fabry disease. Periodic acid–Schiff staining (B, E) and Masson's trichrome staining (C, F) showing expanded glomerular podocytes with fine vacuolization changes in both case 1 (B, C: ×400) and case 2 (E, F: ×400) (bar = 100 μm).

**Fig. 2:**
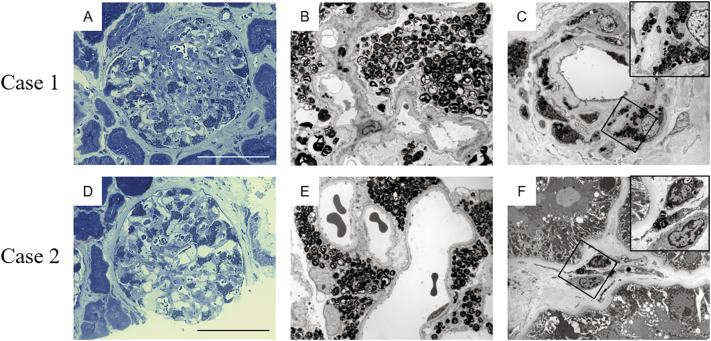
Representative toluidine blue-stained semi-thick scout section (A, D) and electron microscopy images of siblings with Fabry disease (B, C, E, F). Globotriaosylceramide inclusions in a toluidine blue semi-thin stained glomerulus were mainly found in the podocytes (A: Case 1, D: Case 2, ×400, bar = 100 μm). Numerous osmiophilic, lamellated membrane structures with a concentric pattern called myelin bodies were found in the podocytes (B: Case 1, E: Case 2, ×800), in the cytoplasm of the vascular smooth cells (C: Case 1, ×1000, inset: ×5000) and peritubular capillary cells (F: Case 2, ×1200, inset: ×5000).

On admission, the patient's details were: blood pressure 112/72 mmHg, pulse rate 60 beats/min (regular sinus rhythm), height 157 cm, and body weight 63 kg. A chest X-ray showed an enlarged heart shadow without pulmonary edema. Electrocardiography displayed a normal sinus rhythm with normal PR intervals and no dysrhythmias. Echocardiography showed left ventricular hypertrophy with preserved systolic function (left ventricular ejection fraction, 65%). The patient's laboratory data showed that her serum creatinine and blood urea nitrogen levels were 0.51 and 10.6 mg/dL, respectively. Her serum cystatin-C level was 0.69 mg/L (0.48–0.85 mg/L) and creatinine clearance was 138 mL/min (Table 1). Her white blood cell, hemoglobin, platelet, C-reactive protein, blood glucose, and electrolyte levels were all normal. Urinalysis showed no microalbuminuria, normal microscopic findings (urine albumin negative, 10.9 mg/gCre, occult blood negative, and red blood cells 1–4/high power field), and no tubulointerstitial damage (*N*-acetyl-β-D-glucosaminidase 1.3 U/gCr [<8.0 U/gCr], β-2 microglobulin 0.02 μg/mL [<0.22 μg/mL]). Mulberry cells (<1/high power field) were present in the patient's urine (Fig. 1A). Thus, we conducted a percutaneous renal biopsy to investigate the presence of Fabry nephropathy. Unexpectedly, 29 glomeruli showed strikingly enlarged and vacuolated podocytes under light microscopy (Fig. 1B, C), and myelin-like bodies were detected in the podocytes, mesangial, parietal epithelial, proximal and distal tubular, peritubular capillary, vascular intimal, and vascular medial cells by toluidine blue-stained semi-thick scout section (Fig. 2A) and by electron microscopy (Fig. 2B, C). We also found local podocyte foot process effacement in electron microscopy.

**Table 1:** Characteristics and renal findings of siblings with Fabry disease.

	Case 1	Case 2
Age (years)	47	45
Acroparesthesias	+	+
Angiokeratoma	−	−
Cornea verticillata	+	+
Hypohidrosis	+	+
Stroke	−	−
Left ventricular hypertrophy	+	+
Vasospastic angina	+	+
Body mass index (kg/m^2^)	25.6	20.8
α-Galactosidase A (pmol/h/disk)	<1.0	5.7
Urine Protein	(−)	(−)
Urine Occult Blood	(−)	(−)
Urinary red blood cells (/HPF)	1–4	1–4
Urinary mulberry cells (/HPF)	1–4	1–4
UACR (mg/gCre)	10.9	21.0
*N*-acetyl-b-D-glucosaminidase (U/gCr)	1.3	2.6
b-2 microglobulin (μg/mL)	0.02	0.04
Blood urea nitrogen (mg/dL)	10.6	9.0
Serum creatinine (mg/dL)	0.51	0.59
eGFR (ml/min/1.73m^2^)	99.2	85.6
24 h creatinine clearance (mL/min)	138.0	108.0
Cystatin C (mg/L)	0.69	0.67

eGFR: estimated glomerular filtration rate, HPF: high-power field, UACR: urine albumin-to-creatinine ratio.

### 2.2 Case 2: Younger sister

A 45-year-old Japanese woman was admitted together with Case 1. She was diagnosed with Fabry disease based on decreased α-Gal A activity (5.7 pmol/h/disk; cutoff value <7.0 pmol/h/disk in females) and the presence of the same mutation as her brother, c.723dupT in exon 5 of *GLA*. Her clinical course and physical findings were similar to those of her elder sister. Acroparesthesia, hypohidrosis, and cornea verticillata were present but angiokeratomas were absent at the time of diagnosis.

Her details on admission were: blood pressure 122/70 mmHg, pulse rate 60 beats/min (regular sinus rhythm), height 158 cm, and body weight 52 kg. A chest X-ray showed an enlarged heart shadow without pulmonary edema. Electrocardiography displayed a normal sinus rhythm with normal PR intervals and no dysrhythmias. Echocardiography showed left ventricular hypertrophy with preserved systolic function (left ventricular ejection fraction, 60%). The patient's laboratory data showed that her serum creatinine and blood urea nitrogen levels were 0.59 and 9.0 mg/dL, respectively. Her serum cystatin-C level was 0.67 mg/L (0.48–0.85 mg/L) and creatinine clearance was 108 mL/min (Table 1). Her white blood cell, hemoglobin, platelet, C-reactive protein, blood glucose, and electrolyte levels were all normal. Urinalysis showed no microalbuminuria, normal microscopic findings (urine albumin negative, 21.0 mg/gCre, occult blood negative, and red blood cells 1–4/high power field), and no tubulointerstitial damage (*N*-acetyl-β-D-glucosaminidase 2.6 U/gCr [<8.0 U/gCr], β-2 microglobulin 0.04 μg/mL [<0.22 μg/mL]). However, mulberry cells were present (1–4/high power field) in the patient's urine (Fig. 1D). As with her elder sister, we conducted a percutaneous renal biopsy to investigate the presence of Fabry nephropathy. Similarly, 19 of 20 glomeruli showed strikingly enlarged and vacuolated podocytes under light microscopy (Fig. 1E, F), and myelin-like bodies were detected in the podocytes, mesangial, parietal epithelial, proximal and distal tubular, and peritubular capillary cells by toluidine blue-stained semi-thick scout section (Fig. 2D) and by electron microscopy (Fig. 2E, F). We also found local podocyte foot process effacement in electron microscopy as in the case of her elder sister.

### 2.3 The renal pathological findings of two female siblings with Fabry disease

Table 2 shows a summary of the renal pathological scores for both patients. The biopsies were independently scored according to the scoring system of the ISGFN [15], blindly, by one pathologist and one nephrologist who were blinded to the patient diagnoses. Podocyte Gb3 inclusions were scored between 0 and 4 in toluidine blue-stained semithin sections, and podocyte vacuolizations were scored between 0 and 3 in sections stained with periodic acid–Schiff. These scores were combined into a composite score, accounting for all scored glomeruli. The intima and media of arteries and/or arterioles were examined for Gb3 inclusions in toluidine blue-stained semithin sections using a binary system—Gb3 inclusions present or not present. All the 29 scoreable glomeruli in case 1, and 19 of 20 scoreable glomeruli in case 2 showed severe podocyte vacuolization under light microscopy, resulting in mean podocyte scores in cases 1 and 2 of 3.0 and 2.95, respectively (Table 2). Furthermore, the podocyte inclusions scores of semi-thin sections from both patients were 4, which means ≥50% of the tuft was involved with large, expanded deposits. Both patients were initiated on ERT because their genetic mutation was unamenable to migalastat, an oral pharmacological chaperone.

**Table 2:** Patients' scoring sheet for Fabry nephropathy by light microscopy.

	Case 1	Case 2
Podocyte vacuolization in light microscopy		
None (Score = 0)	0	0
Mild (Score = 1:<25%)	0	0
Moderate (Score = 2:25–50%)	0	1
Severe (Score = 3:>50%)	29	19
Total scoreable glomeruli	29	20
Not scoreable (fragment or global sclerosis)	6	8
Total number of glomeruli	35	28
Podocyte score (=n affected x score/total scoreable counted)	3.00	2.95
Inclusions in semi-thin section		
Total number of glomeruli	3	1
Podocyte inclusions score ⁎	4	4
Parietal epithelial inclusions	+	+
Proximal tubular inclusions	+	+
Distal tubular inclusions	+	+
Peritubular capillary inclusions	+	+
Vascular initial inclusions	+	−
Vascular medial inclusions	+	−

⁎ Podocyte inclusions were scored as follows [15]: ‘0’, no deposits; ‘1+’, rare small inconspicuous deposits; ‘2+’, more frequent small deposits; ‘3+’, <50% of the tuft involved with large, expanded deposits in the presence or absence of concurrent small deposits; and ‘4+’, ≥50% of the tuft involved with large, expanded deposits.

## 3 Discussion

We found unexpectedly high pathological scores in two female siblings with Fabry disease presenting with urinary mulberry cells but without microalbuminuria, suggesting that urinary mulberry cells are useful to detect Fabry disease even in patients with minimal or no proteinuria, as their presence may indicate that a significant amount of Gb3 has already accumulated in various cells in the kidney. Therefore, clinicians should consider performing a kidney biopsy to assess the pathological findings in the baseline evaluation of all Fabry patients, even those with mild urinalysis findings.

Mulberry cells and mulberry bodies have been recognized as a useful tool not only for detecting various types of Fabry disease at earlier stages [12] but also for assessing the efficacy of ERT [13]. Selvarajah et al. examined the diagnostic potential of the detection of urinary mulberry bodies in 35 male and female Fabry patients of various clinical spectrums and in 21 control patients with other renal diseases, such as glomerular nephritis or acute tubular injury [14]. The presence of urinary mulberry bodies had a sensitivity of 100% (95%, confidential interval: 85.4–100%) and specificity of 100% (80.8–100%) for the diagnosis of Fabry disease, although repeated and careful examination of urine sediments was required for their detection. In addition, excretion of urinary mulberry bodies was reportedly increased in Fabry patients with renal involvement in a severity-dependent manner [14]. Importantly, mulberry bodies can be detected even in women with heterozygous Fabry disease with normal renal function and no albuminuria [14], as with our sibling cases.

In the present report, we describe renal histological findings in two female siblings with classic Fabry disease, prior to urinary protein loss (albuminuria/proteinuria). Most of the glomeruli received a score of 3 in the ISGFN scoring system [15] due to the presence of severe podocyte vacuolization. This suggests that glomerular and vascular changes are present before progression to overt proteinuria and decreased glomerular filtration rate, as reported by Tøndel et al. [17]. It has also been reported that podocyte foot process effacement is an early marker of nephropathy in young, classic Fabry patients without albuminuria [18], as observed in our cases. For this reason, renal biopsies are useful for the diagnosis of Fabry disease, as well as to determine treatment efficacy in follow up, although careful consideration should be given to the patient's particular case.

We recently reported that simple screening by analyzing α-Gal A activity in dried blood spot samples is useful for the early diagnosis of Fabry disease in high-risk and underdiagnosed patients. The diagnosis of Fabry disease was made in four men and two women who had different pathogenic *GLA* mutations (0.26%) and four men who had the *GLA* c.196G > C (p.E66Q) variant (0.17%) among 2325 patients who suffered from various cardiac, renal, or neurological manifestations [5]. Given this, the combination of measuring α-Gal A activity in dried blood spots and screening for urinary mulberry cells and mulberry bodies might be very useful in detecting Fabry disease at an early stage. At present, there is no evidence that urinary mulberry cells and mulberry bodies have a direct role in the pathogenesis of kidney damage. Although Gb3 accumulation might be the first step in Fabry pathophysiology, followed by the reviewed biological processes that contribute to tissue inflammation and fibrosis [11], further studies are required to investigate the role of urinary mulberry cells and mulberry bodies in the pathogenesis of Fabry nephropathy.

In this case report, kidney biopsies provided useful information for the diagnosis of Fabry nephropathy, confirming the clinical suspicion. Although urinary mulberry cells and bodies are promising early biomarkers of Fabry disease, a kidney biopsy is a necessary intervention to assess the pathological findings in the baseline evaluation of all Fabry patients, even without microalbuminuria. Future development of a chronicity pathological scoring system, based on longitudinal data and virtual slide libraries, may be useful for describing the severity of pathological changes and their prognostic implications for progressive Fabry nephropathy patients.

## Declaration of Competing Interest

None.
